# Nexus between information and communication technologies and life expectancies of low-income countries: Does technological advancement increase their life span?

**DOI:** 10.1016/j.ssmph.2023.101600

**Published:** 2024-01-06

**Authors:** Wenxin Wang, Issam Khelfaoui, Danish Ahmed, Yuantao Xie, Muhammad Hafeez, Hicham Meskher

**Affiliations:** a School of Public Health, Shantou University/Institute of Local Government Development, Shantou University, Shan-Tou 515063, China; b Department of Operational Research, Faculty of Mathematics, University of Science and Technology Houari Boumediane, China; c School of Finance and Economics, Jiangsu University, Zhenjiang, Jiangsu 212013, China; d School of Foreign Language, Shanghai Jianqiao University, Shanghai 201315, China; e Department of Business Administration, HANDS—Institute of Development Studies (HANDS-IDS), Karachi 75230, Pakistan; f Center for Islamic Finance, University of Bolton, Bolton BL3 5AB, UK; g International Institute on Governance and Strategy (IIGS), Beijing 100000, China; h School of Insurance and Economics, University of International Business and Economics, Beijing 100029, China; i Institute of Business and Management Sciences, University of Agriculture, Faisalabad, Pakistan; j Chadli Bendjedid El Tarf University, Eltarf, Algeria; k Adnan Kassar School of Business, Lebanese American University, Beirut, Lebanon

**Keywords:** Low-income countries, Information technology, ICT, Life expectancy

## Abstract

Access to state-of-the-art infrastructure is inevitable for a higher standard of living for the people of any country. At least, this has been the case for developed countries. This study investigates the link between information and communication technologies (ICT) and life expectancy at birth (LEB) among low-income countries. We use panel data of low-income countries from 2000 to 2017 from the comprehensive World Bank dataset. Our analysis strategy includes employing Driskol and Kraay methodology and feasible generalized least squares to tackle cross-sectional dependence. Furthermore, we also employ the instrumental variable technique to deal with the endogeneity problem. We found that a rise in mobile internet use and Mobile Cellular Subscriptions led to improved LEB among low-income countries. On the contrary, the rise in fixed telephone subscriptions had a negative empirical effect on reducing LEB—however, the magnitude of the effect ranged between 0% and 4%.

## Highlights

• This research used the longest panel data study available on LICs regarding ICT impact on health outcomes.
• This research used the PMM to complete the data and estimated with Driscoll and Kray models with IVs.
• This research also used feasible generalized and Common Correlated Effects models.
• Internet Use and Mobile Cellular Subscriptions elevate Life Expectancy at Birth of low-income countries.
• Fixed Telephone Subscriptions decrease the Life Expectancy at Birth health outcomes of low economies.

## 1 Introduction

Everyone should have the right to access healthcare services. The United Nations believes that keeping people healthy is essential for a country's long-term success. However, in low-income countries, there often needs to be more hospitals and clinics, which makes it harder for people to get the healthcare they need. The former issue is causing big problems for these countries, like shorter lifespans than more higher income countries. A report by the World Health Organization also said that only some have equal access to healthcare, which is one of the reasons why there are significant differences in how long people live. Accessibility to the best hospitals and equipment makes getting healthcare in any country easier. In this study, we are looking at how technology can help improve healthcare in lower income countries. We are using life expectancy to measure how healthy people are and technology to measure how advanced a country is. If health technology improves than it is currently, will people in low-income countries get healthier?

People are now living longer all around the world. In 2000, the average age a person could expect to live was 66.5 years, but by 2016, it had increased to 72 years. This outcome means that people are living for longer expectancies. Not only are people living longer, but they are also living healthier lives. In 2000, the average number of healthy years a person could expect to live was 58.5, but by 2016, it had increased to 63.3 years. However, higher and lower-income countries still have a big difference in life expectancy compared to lower-income ones. On average, people in rich countries live 18.1 years longer than people in low-income countries. The previous difference in health disparities is because people in low-income countries need more access to good healthcare. Everyone needs access to good healthcare to live long and healthy lives. The United Nations has set a goal to ensure everyone can access good healthcare. However, in low-income countries, healthcare is not as good as in rich countries (Bokhari et al., 2007; Papa, Mital, Pisano, & Del Giudice, 2020). Despite the efforts and strategies discussed by the UN assembly, health still needs more work and effort to be elevated. The global health situation “Health outcomes" for low-income countries will continue to worsen, and the health gap between them and higher-income countries will widen if they do not control for the real causes and implement further measures to reduce them. Many propositions have been put forward to achieve the previous goal. This paper will analyze one of those propositions. Specifically, this research paper focuses on whether ICT elevates health outcomes in low-income countries by measuring the causal effect of ICT variables on life expectancy at birth.

ICT for health is a big money-making market. People are getting better at using technology and staying connected. Therefore, lower-income countries want better healthcare and more access to technology. Governments want to employ technology to make healthcare cheaper and easier to get. This governmental approach creates many chances to make new technologies for healthcare (Flick, Zamani, Stahl, & Brem, 2020).

Based on numerous health theories, including the Health Belief Model, the Transtheoretical Model and Stages of Change, Social Cognitive Theory, and the Social Ecological Model, a Theoretical relationship between health and ICT exists. It has been shown that interventions with a clear conceptual framework are more efficient than those with an ambiguous. Moreover, tactics that mix numerous ideas and concepts have a more significant impact. Different treatments should positively affect individuals and interpersonal, organizational, and environmental factors to influence health status. Furthermore, this can only be achieved via efficient and continued ICT use for health.

The advancement and development of ICT can improve health outcome indicators by bringing the beneficiaries and providers of healthcare closer and reducing their related geographical constraints (Nasir & Kalirajan, 2016). ICT is a strong pillar of modern infrastructure in this new millennium of globalization. This pillar brings critical innovations to all facets of life, economy, and especially health; it plays a valuable role in developing the socio-economic aspect of societies. In contrast, lofty and numerous research articles validate the positive effect of ICT on GDP, poverty alleviation, capacity or economic development, and employment (Broersma & Van Ark, 2007; Dabinett, 2002; Pradhan, Arvin, Norman, & Bele, 2014). There are even more publications on the relation between the wheel of innovation and practical information spread and ICT, where ICT is a practical driving force forward for this economic wheel (Lee & Brahmasrene, 2014). The studies above show four principal paths of how ICT drives socio-economic development forward. These paths are business retention, competitiveness, economic diversification, and quality of life (Lee et al., 2012, 2016).

The importance of offering effective and efficient ICT for a healthy lifestyle is rising (Talukder, Sorwar, Bao, Ahmed, & Palash, 2020). However, it is only sometimes apparent how or if ICT developers and creators consider the human repercussions of their technology-related goods and services. Technology is frequently implemented in manners that exacerbate present healthcare inequalities and put people on the brink of harm. Health ICTs tend to be seen as “remedying health circumstances” rather than “life support” (Ballegaard, Hansen, & Kyng, 2008). A thorough literature review revealed a need for more empirical studies addressing the effects of technology use on health. Prior studies have focused on how ICTs affect patients with long-term illnesses, dietary assessments, and physical activity. This study found that ICT can affect various health metrics in good and bad ways. A different area of earlier study emphasizes ICTs' usability from the perspective of young and middle-aged adults (Talukder et al., 2020; Gao, Prina, Wu, & Mayston, 2022; Gao et al., 2022b). These academic articles, however, came under criticism for not considering other data sources and for looking at regression coefficients utilizing conventional statistical methods.

Taking the importance of the driving force of ICT, in this study, the authors aimed to empirically address this difference gap using the proper cross-sectional panel data approaches on selected low-income countries from 2000 to 2017.

### 1.1 The contributions

In many ways, the paper contributes significantly to the latest studies. To begin, it used extended cross-sectional models to comprehend and forecast the effects of various ICTs on LIC life expectancy and outcomes. Second, it expanded the use of the cross-sectional panel unit roots test to include theoretical constructs. It is thirdly, using a data mining approach to bridge the gap of data loss. The cornerstone of this study recognizes the validity and effectiveness of traditional statistical methods, as earlier research and work have established the foundation for reinterpreting results from a newly cross-sectional predictive perspective. The paper outlines the broad ramifications and their implications for governments and policymakers. Therefore, the present study pinpoints the existing research gaps by formulizing cross-sectional panel data models to predict ICT effects on lower-income countries. The question we wish to address is what technical developments in ICT for health might be positive and what policy advancements are necessary to implement. Addressing this query is extremely useful since it offers the foundation for technology developers and businesses to appropriately place their goods and services. Because of these technologies' substantial operational influence, understanding future technologies and their ethical and social ramifications is crucial for policymaking. This study also serves as a precursor to extensive empirical research in broad application areas for ICT for countries' health.

Following the introduction, this research applied an empirical methodology to reach the goal. The paper is structured as follows: Section 2 proposes the theoretical related literature backbone of the paper regarding ICT and health. Section 3 provides this study's data curation and methodology, explaining the empirical necessary panel data tests. Results and discussions are presented in Section 4. In conclusion, Section 5 emphasizes a substantial discussion and identifies three subsections for concluding remarks, managerial and theoretical implications, and limitations with issues for upcoming research.

## 2 A brief literature review on ICT and health outcome

The present study focused on the effects of ICT on health outcomes in low-income countries. Therefore, the null hypothesis to be tested was:

H0 ICTs do not have any effect on the health outcomes in low-income countries.

### 2.1 The literature on the positive effect of ICTs on health outcomes

ICT breaks down spatial or temporal information exchange barriers with trivial cost and noticeable efficiency (Kaplan, 2006). This function is highly prevalent in all facets of the socioeconomic axis, especially the health axis, and many types of research well document it. Individuals, particularly patients, now have easy access to a wealth of health-related information thanks to the advancement of ICT and the Internet. This ease of entry enables effective and efficient communication and time with physicians. It betters health decision making for patients, and it eases possibilities for functional health interplay and healthcare services implementation (Ball and LillisE-health, 2001; Broom, 2005). Bukachi and Pakenham-Walsh (2007) refer to the fact that healthcare workers use the internet for correspondence, access to valuable clinical information, and global collaboration. Wald, Dube, and Anthony (2007) and Bankole, Osei-Bryson, and Brown (2013) provide an excellent overview of the relationship between healthcare and the internet as well as the doctor–patient relationship. According to their survey findings, the internet has the potential to improve health structures and global health.

Lucas (2008) considers the four potential promising areas for developing economies to investigate the health consequences of ICT innovations. These four areas are ICT systems, computer-assisted prescription monitoring, treatment, and diagnostics, a wide range of applications labeled “telemedicine”, and the facilities of ICTs. Taking Uganda's pregnant women's healthcare case, a project on radio technology reduced the maternal mortality rate (Makar, McMartin, Palese, & Tephly, 1975). Another successful example is the Bangladesh “Mobile for Health” project. This mobile health project shifted the rate of childbirths with measurable success. Where originally, 90% of child deliveries took place outside of hospitals, 89% of children delivered inside hospitals (Schmoldt, Benthe, & Haberland, 1975).

Blaya, Fraser, and Holt (2010) reviewed the developments of e-health-applying information technologies to manage patient care fulfillment for the same developing economies. They provide evidence that e-health has a real impact on health in developing countries. They claim that ICT systems improve interactions among institutions, aid in medication organization and distribution, and promote monitoring and identifying patients who may forego or drop out of care. Furthermore, mobile and personal digital appliances speed up and improve the quality of data acquisition. Cole-Lewis and Kershaw (2010) present a systematic review of telephone text messaging as a tool for changing individuals’ behavior toward disease management and illness prevention. They see encouraging evidence for diabetes control, smoking cessation, and weight loss. They claim that text messaging can be an effective tool for reducing global healthcare responsibility by providing more practical disease prevention and control.

Furthermore, Déglise, Suggs, and Odermatt (2012) examined contextual studies from developing countries to investigate SMS interventions for communicable and noncommunicable illness and disease prevention, monitoring, supervision, and treatment acquiescence. They present evidence that cell phones can be used to improve disease control in developing countries. They discuss how cell phones are a cost-effective way to address the demands and lack of healthcare systems, and how they can create unusual opportunities to improve the global health of developing countries. Nilseng et al. (2014) discovered that ICT tools can improve stock management and medication ordering among healthcare operators in a pilot study conducted in Tanzania between 2010 and 2011.

The use of mobile technology and nowadays the Internet is highly underestimated, whether in earlier years of text messages for different vaccinations distribution campaigns, currently with the ease of access to numerous health-related information sources, or the fast acquaintance with new diseases and how to take protective measures such as the case the world faced with the COVID-19 virus in 2020. Another noticeable study design by Fedha (2014) found that ICT significantly raised pregnant women's attendance rates at clinical appointments which, in turn, reduces children's mortality rates. On the other hand, ICT reduced pregnant women's unnecessary attendance rate at clinical visits due to the ease of communication and information diffusion. The development and innovation of communication tools and applications through ICT helped and has helped create a real-time feedback system. This real-time information diffusion system decentralizes the entirety of the healthcare system for many regions in the world (Lacal, 2003; Mechael, 2007).

Moreover, any development in any system needs adherence to all the innovations and information available in all scientific disciplines. Some of these disciplines are computer science, education, psychology, and chemistry. The exponential growth of the adherence of these disciplines through their projection of information aids in providing better health results for patients, aiding in the development and the resolution of vaccination problems, which researchers noticed in more than 15,000 published studies just on COVID-19 over the past six months (Neuhauser & Kreps, 2003; Noordam, Kuepper, Stekelenburg, & Milen, 2011). This exponential growth guarantees a revolution in the sector of health through ICT development. Such a revolution has been noticed in many areas of the world. Taking the example of Uganda, this revolution saved more than 25% of the country's health expenditure, just through the massive reduction of their data collection and storage costs (Valk, Rashid, & Elder, 2010). From the cost perspective, ICT are cost-efficient and effective. This enables many individuals to exchange and share health-related experiences that reduce the effect of common health issues (Ballegaard et al., 2008). Finally, ICT is a great tool, as illustrated by many studies, that helps the elderly self-manage in a better way, individuals interact with their health concerns, and eases communication between the elderly or sick ones with their families, and many other uses for this great tool (Ball and LillisE-health, 2001; Wilson, 2004a). ICT aids in self-supervision measures by facilitating communication tools for senior citizens, making it far easier for them to contact their children, family members, and physicians (Ball and LillisE-health, 2001; Wilson, 2004a, 2004b, 2004c).

Recent research has shown that ICT can improve the indicators of well-being by reducing geographical barriers and bringing medical care providers and recipients closer together (Nasir & Kalirajan, 2016). Cole, Watkins, and Kleine (2016) investigate the nature of health advice obtained from web discussion blogs and forums. Their investigation revealed little evidence of poor-quality health advice and information. They proposed that discussion blogs and sites could be a good platform for people to ask health-related questions and receive quality responses. Tsai, Chao, Lin, and Cheng (2018) investigated the ‘‘blend e-learning system (BELS)’’ of Taiwanese nursing personnel and discovered that its e-learning curriculum joined “face-to-face” courses in advanced health education. Majeed and Khan (2019) use panel data analysis to examine the relationship between ICT and health outcomes in 148 countries from 1990 to 2014. They established that ICT improves global health outcomes by increasing life expectancy and decreasing infant mortality, and they advocate for healthcare plans and policies that prioritize digital inclusion.Dutta, Gupta, and Sengupta (2019) studied the long-run impact of the ICT index on health outcomes using panel data from 30 Asian countries from 2000 to 2014. Their findings confirmed that ICTs had a significant impact on health in the 30 Asian countries. From the previously mentioned literature, a health economist may deduce the first alternative hypotheses, which is the following:

H1a ICT affects the health outcome of low-income countries positively.

### 2.2 The negative effect of ICTs on health outcomes’ related literature

Aside from the positive effects of ICT on health outcomes, there are also negative opposing effects of ICT on health. The first one is the quality of information that ICT may make available online. Information of poor quality may be misleading or misinterpreted. It has the potential to jeopardize health behaviors and outcomes. According to Kiley (2002), false information can cause fear and worry regarding preventable illnesses or death. The second point is that, even in advanced economies, there are still disparities in Internet access between socioeconomic groups (rural versus urban groups, wealthy groups versus unprosperous groups). This problem on a global scale may widen the gaps between these groups, complicating an already existing inequity problem. Third, health information leads to an increase in medical visits, which is a waste of their time and the time of physicians (Murray, Cooper, Wilson, & Romaniuk, 2003).

Bend, Morawczynski and Ngwenyama (2007) argue that low-income countries should prioritize health infrastructure and freshwater rather than ICT-developed tools for improving health behaviors and systems. Tanis, Hartmann, and tePoel (2016) study indicated that health anxiety channels a strong desire to explore and find health information online; however, this strong desire is negatively associated with content geared toward doctors’ comfort.

The key obstacle in maximizing the benefits of ICT in emerging economies is its “implementation”. Aside from a lack of human-trained personnel and adequate human resources to effectively implement, there are four implementation costs to consider: licensing fees, yearly or monthly upgrading costs, subscription fees, and replacement expenses. Moreover, the literature debates that significant reliance on foreign sources creates an unnecessary dependency cost in the long run. Moreover, the harsh climate of the various developing countries damages ICT hardware and instruments that require a climate-supervised setting and dust-free conditions. From the previously mentioned literature, health economists may deduce a second alternative hypothesis, which is the following:

H1b ICT affects the health outcome of low-income countries negatively.

In recent years, policymakers and researchers have focused heavily on health outcomes associated with ICT and ICT infrastructure. However, there are theoretically contradictory results between health and ICT, particularly for low-income countries. An empirical evaluation of the link between health and ICT for low-income economies is required for a more in-depth understanding of the relationship. Previous empirical evidence of the relationship between health and ICT schemes is frequently based on clinical records or specific country records and cannot be generalized to different samples. In the light of the above considerations, this paper aimed to tackle the gap in research in many ways: First, the literature gap explaining the real effect of different ICT variables on health outcomes (measured by life expectancies at birth) across low-income countries. Second was the data gap, different from previous studies, robust data predictive techniques are used, which allowed us to utilize more years and more countries for all the three ICT variables. Third, in the data set, the appropriate cross-sectional data tests, such as cross-sectional panel unit-root tests, are utilized to distinguish the difference in our data from the previous data sets. Fourth, proper models and techniques were applied to deal with the cross-sectional aspect of our data. Finally, novel instrumental variables (IVs) are proposed to robust check our results. The results of this study could help in the formulation of public policy regarding the improvement of health outcomes in low-income countries.

## 3 Data and methodology

### 3.1 Data

Following the lead of previous studies, the authors acquired all the data from the World Bank database. As the aim was to focus on low-income countries, the World Bank defines low-income countries as nations that have a per capita gross national income (GNI) of less than $1026. In this regard and to the present moment, there are exactly 28 countries (i.e., Afghanistan; Burundi; Benin; Burkina Faso; Central African Republic; Congo, Democratic Republic; Eritrea; Ethiopia; Guinea; Haiti; Liberia; Madagascar; Mali; Mozambique; Malawi; Niger; Nepal; Korea Democratic People's Republic; Rwanda; Sierra Leone; Somalia; Syrian Arab Republic; Chad; Togo; Tajikistan; Tanzania; Uganda; and the Yemen Republic).

#### 3.1.1 The dependent output variable

In this research, the dependent variable that represents health outcome is measured through life expectancy at birth, total (years) LEB (Cutler et al., 2006; Adeola & Evans, 2018; Alhassan & Adam, 2021; Bankole & Mimbi, 2016; Bhusal, 2022; Jing, Ma, Zhang, & Hafeez, 2022; Karaman Aksentijević, Ježić, & Zaninović, 2021; Lee et al., 2016; Majeed & Khan, 2019; Văidean & Achim, 2022).

#### 3.1.2 The independent input variable

The most widely used aggregate ICT indicators in literature are individuals using the internet (% of the population) IU, fixed telephone subscriptions (per 100 people) (FTS), mobile cellular subscriptions (per 100 people) (MCS), fixed-broadband subscriptions for the 100 inhabitants FBS, and secure servers are servers used per a 1 million populations (SIS). In Table 1, the gap produced by the data use of other related literature is presented briefly. Following the previous literature that linked ICT to LEB, this paper focused on the first three indicators IU, FTS, and MCS.

**Table 1:** A brief illustration of the data usage gap produced by the relative literature.

Reference/panel specifics (time span and number of countries)/ICT indicator	The data gap in research
184 countries spanning over 1990–2014, IU, FTS, and MCS (Majeed & Khan, 2019)	- short and old time series -their focus was all available countries' data
131 middle income countries spanning over 2005–2018, IU, FTS, and MCS (Văidean & Achim, 2022)	-short time series -their focus was middle income economies
27 African countries spanning over 1998–2007, IU, FTS, and MCS (Bankole & Mimbi, 2016)	-short time series -their focus was on African economies
49 African countries spanning over 1990–2015, FTS and MCS (Bhusal, 2022)	-short time series -their focus was on African economies -only two indicators used
18 Asian countries spanning over 2007–2019, IU (Jing et al., 2022)	-short time series -their focused was on Asian economies -only one indicator used
121 countries only for the year of 2018 IU, MCS (Alhassan & Adam, 2021)	-absence of time series -their focus was on European and Asian economies -only two indicators used
61 countries spanning over 2000–2009 IU, FTS, MCS (Lee et al., 2016)	-short time series -their focus was randomly selected countries

IU is internet use; FTS is fixed-telephone subscriptions, and MCS is mobile cellular subscription.

#### 3.1.3 Control variables

According to the previous literature, there are three types of control factors: economic, social, and environmental factors. The economic controls could be defined by: GDP per capita, PPP (constant 2017 inter ratio in USD), physicians (per 1000 people), the age dependency ratio (% of working-age population), immunization against measles (% of children ages 12–23 months), immunization against DPT (% of children aged 12–23 months), domestic general government health expenditure per capita, PPP (current inter ratio ln USD), and current health expenditure per capita (current USD) (Dutta et al., 2019; Lee et al., 2016; Majeed & Khan, 2019).

The social factors could be defined by sex ratio at birth (male births per female births), secondary school enrollment (% gross), female pupils as a percentage of total pupils at the primary level including enrollments in public and private schools, and the net primary school enrolment rate (%) (Khelfaoui et al., 2022; Lee et al., 2016; Majeed & Khan, 2019; Morawczynski & Ngwenyama, 2007).

The environmental variables could be defined by the urban population (% of the total population), people using at least basic drinking water services (% of the total population), the percentage of the population with access to an improved water source, and CO2 emissions (metric tons per capita) (Khelfaoui et al., 2022; Lee et al., 2016; Majeed & Khan, 2019; Morawczynski & Ngwenyama, 2007).

#### 3.1.4 Instrumental Variables

Different from the previous literature, the following instrumental variables are proposed: Communications, computers, etc. (% of service exports/imports, balance of payments (BoP)), ICT service exports (% of service exports, BoP), ICT goods imports/exports (% total goods imports/exports), computer, communications, and other services (% of commercial service imports/exports). A dummy variable of the first year a country issued an internet country code was also proposed (Khelfaoui et al., 2022).

### 3.2 Data processing

In the processing phase of our data, the authors went through five steps which are illustrated in Table 2.

**Table 2:** A step-by-step description of the actions preformed for data processing and variables selection.

The step definition	The action performed
Step 1: “First leg variable choice”	In this initial step, only the variables that were mentioned in the previous literature are favoured
Step 2: “Timespan”	The period for this study is selected and decided upon according to availability. Given that both data before 2000 and data after 2017 were not available for our variables of interest (ICT) or missing, only the period of 2000–2017 is included, while the other periods are excluded
Step 3: “Second leg variable choice”	Excluding the variables that were completely or partially missing for the 28 countries—exactly the variables that exceeded the 20% missing rate
Step 4: “Country selection”	Excluding the countries with many missing variables or unrealistic values for our variables of interest, e.g., “Korea Democratic People's Republic”
Step 5: “Data imputation”	Completing the missing data with a rate of “7.5%” through the predictive mean matching (PMM) technique. The strength of any imputation technique is greater with more information you provide for it as an entry. PMM imputes and completes missing continuous values in data sets. PMM attributes a present observed value that is nearest to the missing case predicted one. PMM preserves the initial distribution of the experimental data better than fully parametric multiple imputation strategies and is known to be consistent and robust for a small percentage of missing values (Kleinke, 2017; Van Buuren, Brand, Groothuis-Oudshoorn, & Rubin, 2006)

In the end, the data processed had 27 low-income country's data for an 18 year span with the included variables which are summarized in the descriptive statistics (Table 3).

**Table 3:** Descriptive statistics of the dependent, independent, control, and instrumental variables.

Symbol	Descriptive statistic				
	mean	SD	min	max	Median
Dependent variables					
LEB	57.67	6.669	39.44	74.43	57.66
Independent Variables					
IU	4.561	6.519	0.004	34.253	1.549
FTS	1.528	3.307	0.005	22.620	0.504
MCS	28.685	29.752	0.018	138.80	20.008
Control variables					
GDP	546.1	330.13	111.9	2032.6	467.7
SCH	93.17	29.436	16.63	156.03	92.38
IM	69.38	18.056	16.00	99.00	70.00
IDPT	70.31	19.570	19.00	99.00	74.00
HE	30.242	19.067	4.691	139.75	24.814
ADR	90.09	11.710	56.61	111.94	91.06
SR	1.038	0.014	1.010	1.071	1.030
UP	29.744	11.374	8.246	55.60	29.909
CO2E	0.246	0.497	0.017	3.343	0.0947
Instrumental variables					
ICC	0.277	0.448	0.000	1.000	0.000
CCSEB	6.988	6.408	0.144	42.219	5.631

SD is the standard deviation; Min is minimum; Max is the maximum; LEB is life expectancy at birth; IU is internet use; FTS is fixed telephones; MCS is mobile subscriptions; GDP is GDP per capita; SCH is school enrollment, secondary (% gross); IM is immunization against measles; IDPT is immunization against DPT; HE is domestic general government health expenditure per capita; PPP (current interratio ln USD); ADR is age dependency ration; SR is sex ration; UP is urban population; CO2E is carbon oxide emissions; ICC is internet country code dummy; and CCSEB is communications, computer, etc. (% of service exports, BoP).

### 3.3 R/RStudio programming tools

In this paper, the authors utilized many R packages to perform the statistical technical data processing testing and data modeling. To handle the data, process it, and apply the PMM technique, the “MICE” package was utilized within programming commands. For the graphing, the “ggcorplot” and “gplots” packages were utilized. Data testing and data modeling were handled through “plm”, “punitroots”, and “car”. All the packages are openly accessed in R and any coding was performed to ensure the smooth application of the methods on the data.

### 3.4 Empirical tests

To utilize the model described beforehand and to evaluate the robustness of the results a set of tests has been used. The tests that were used, which are widely common in literature, were:

Panel unit root test: To test for stationarity properties, there are two generations of panel unit roots. The first-generation tests commonly used: Levin–Lin and Chu (LLC) and Im–Pesaran–Shin (IPS) tests (Im, Pesaran, & Shin, 2003; Levin, Lin, & James Chu, 2002). Both tests are based on the augmented Dickey–Fuller unit root test, and both have similar null hypotheses. Their null hypothesis indicates no presence of unit roots. The second-generation tests are distinguished by the rejection of the cross-sectional independence assumption. These tests are cross-sectional Im–Pesaran–Shin CIPS and cross-sectional augmented Dickey–Fuller augmented Dickey–Fuller CAFD (Pesaran, 2007). In this endeavor, the authors started first with preliminary first-generation tests to set a prior assumption on the data. After, the application of the estimation methods and the following set of tests on their estimates, the presence of cross-sectional dependence was established. When the assumption of no cross-sectional dependence was rejected, then the second-generation tests of unit roots under cross-sectional dependence were applied.

Panel cross-dependence tests: Inspection of the presence of unmeasured unit cross-dependence in the model estimation, which in our paper was the presence of country cross-dependence (Breusch & Pagan, 1980; Pesaran, 2021).

Hausman model specification test: It is commonly used to specify the estimation method that explains better heterogeneity in models. Usually, it is used to distinguish which model estimates are better, its null hypothesis indicates random-effects model estimates that are more consistent, while the alternative indicates the fixed-effect estimates that are more consistent (Hausman, 1978).

### 3.5 Model construction

In Grossman's (Grossman, 1972) Health Production Function, health is produced by individuals depending on their comportment, medical care, and the limited resources they have. Theoretically, Grossman's (Grossman, 1972) production of health function is given in (1):

(1) Health=f(healthinputs)

where “Health” is a person's output of health, and health inputs are the following factors: income and wealth, education level, health expenses and investments, health accommodations, milieu, and the standard of living. “Grossman's” model studies the health production function microlevel (Grossman, 1972). Fayissa and Gutema (2005) converted it to its equivalent macrolevel. They re-expressed health inputs per capita and reconstructed them in three divisions: economic, environmental, and social. Therefore, their updated utility demand for health function is presented in (2):

(2) Health=f(ECO,ENV,SOC)

where “ECO”, “ENV”, and “SOC” describe factors of economic, environmental, and social variables, respectively. There are numerous and different variables that come under each division. Thus, every research has adopted different variables due to data and resource limitations or other reasons.

In our empirical research, the economic factors were economic growth (GDP and ADR) and health expenditures (HE, IM, and IDPT), the social variables were education SCH, sex ratio (SR), and the environmental factors included urban population ratio (UP) and carbon dioxide emissions (CO2E). This is illustrated in Eq. (3) as follows:

(3) Health=f(Economicgrowth,Healthexpenditures,SCH,SR,UP,CO2E)

This research explored other potential factors that may affect health by concentrating on ICT. To consider the effect of ICT on the production of health models, Majeed et al. (Majeed and Khan, 2019b) prolonged Eq. (3) to incorporate digital inclusion. This resulted in Eq. (4):

(4) Health=f(Economicgrowth,Healthexpenditures,SCH,SR,UP,CO2E,ICT)

In this study, ICT was measured through fixed-telephone subscriptions (FTS), mobile cellular subscriptions (MCS), and internet use (IU). As follows, health was quantified through LICs' life expectancies at birth.

Dutta et al. (2019), Majeed and Khan (2019), the above equation between “ICT and Health” and our model can be used and taking into consideration the ease to build a link between them through using log functional form and interpreting the coefficients, formulated to the panel equation design (5).

(5) $${\text{lnHealth}}_{\text{it}}=\alpha_{\text{it}}+\beta_{1}{\text{lnICT}}_{\text{it}}+\beta_{2}{\text{lnX}}_{\text{it}}+\varepsilon_{\text{it}}$$

Here, i is the country index, and t is the time yearly index. Health represents the dependent variable in our design measured by LEB. ICT represents the independent variable in our design measured by IU, FTS, and MCS. X represents the set of control social, economic, and environmental factors mentioned before and measured by GDP, SCH, SR, IM, IDPT, HE, ADR, UP, and CO2E. “ln” is the natural logarithm function. ε indicates the idiosyncratic error term. To estimate the panel model parameters, the authors first applied pooled ordinary least squares (POLS), and then random- and fixed-effects (RE/FE) models. $\alpha_{i}$ had three forms: constant $\alpha_{0}$ for the POLS model; country, time and two-way (i.e., time and country) fixed effects $\alpha_{i}$, $\alpha_{t}$, and $\alpha_{\text{it}}$ in the fixed-effects model; and constant and random effect errors $\alpha_{o}{+\alpha }_{\text{it}}$ in the random effect model.

In dealing with the cross-sectional dependence issue, the authors estimated the three regression models (i.e., POLS, RE, and FE) with Driscoll and Kraay standard errors. In the presence of cross-sectional dependence, the standard errors of Driscoll–Kraay are well calibrated (Driscoll & Kraay, 1998; Hoechle, 2007). To deal with the observed and unobserved cross dependence in the data (i.e., when the regressors and errors both have a factor structure), the common correlated effects (CCEs) is the appropriate modeling approach. This approach has become very influential in the theoretical panel data literature and empirical applications. The CCE method presented by Pesaran (2006) and developed by many authors, including Bai (Panel data models with interactive, 2009), consists of an equation of interest in which the error has a component structure and a reduced form equation. In this form, the explanatory variables are linear functions of the same factors, which are present in the main equation. Then, the CCE treats the response and explanatory variable cross-sectional averages as fixed effects, removing unobserved variability. The latest paper presented by Juodis, Karabiyik, and Westerlund (2021) delivers strong evidence of the robustness of the pooled CCE. Therefore, the authors also applied the pooled PCCE with Driscoll and Kraay's robust errors. Finally, three factors are likely to cause endogeneity in our model. The first factor was the simultaneous link between health and ICT factors. The second factor was present in ICT measures that correlated with error terms. The third factor was the issue of omitted variable bias. These issues arise for various reasons, but they all have a workable approach: the use of instrumental variables. This study employed two-stage least squares in the cross-sectional data (both FE and FGLS) to resolve the endogeneity issue. The potential endogenous variable (ICT) was instrumented with several appropriate internal and external instruments. The instruments used are communications, computers, etc. (% of service exports, BoP), and internet country code's dummy. The indicators of communications, computers, etc. (% of service exports, BoP), and internet country code dummies correlate strongly with ICT measures. Furthermore, these indicators have no direct influence on population health. The data for instrument variables are in Table 4’s instrument variables part.

**Table 4:** Panel unit root tests.

Symbol	Second-Generation Panel Unit Root test				First-Generation Panel Unit Root test	
	CIPSd	CAFDd	CIPSt	CAFDt	LLC	IPS
LEB	−1.452*	−14.528*	−7.218*	−2.329*	−11.71*	−11.59*
IU	−2.478*	0.225	−3.475*	−2.649*	−6.103*	−0.749
FTS	−1.6*	0.48	−1.886*	2.172*	−5.01*	−0.627
MCS	−3.732*	−8.144*	−4.002*	3.614*	−23.233*	−17.663*
GDP	−2.487*	−1.587*	−2.731*	2.29*	−4.717*	−1.097
SCH	−2.592*	−3.943*	−2.744*	−2.98*	−6.797*	−4.754*
IM	−2.571*	−3.902*	−2.608*	−1.873*	−6.758*	−5.357*
IDPT	−2.268*	−5.042*	−2.702*	−3.116*	−13.213*	−9.030*
HE	−2.138*	−3.262*	−2.406*	2.278*	−5.633*	−2.265*
ADR	−0.497	4.937	−0.677	5.694*	3.023	15.379
SR	−2.895*	−8.99*	−3.213*	−8.27*	−3.9749*	−10.594*
UP	−1.017	5.255	−2.343*	−1.728*	−24.437*	−5.148*
CO2E	−1.911*	−1.293	−2.05*	1.455	0.777	−1.748*

d: stands for drift; t: stands for trend; Significance level, *p < 0.1. LEB is life expectancy at birth; IU is internet use; FTS is fixed telephones; MCS is mobile subscriptions; GDP is GDP per capita; SCH is School enrollment, secondary (% gross); IM is immunization against measles; IDPT is immunization against DPT; HE is domestic general government health expenditure per capita; PPP (current inter ratio ln USD); ADR is age dependency ration; SR is sex ratio; UP is urban population; CO2E is carbon oxide emissions; ICC is internet country code dummy; and CCSEB is communications, computer, etc. (% of service exports, BoP).

## 4 Results

In this research, the authors report the results in eight parts. The first part envelopes a broad description of the data, their Spearman correlation, and unit root test results. The 2nd to the 4th parts report individual and two-way FE with and without IV, pooled and mean-group CCE estimation results, and FGLS estimation results, respectively. As per the cross-dependence test and Hausman model specification test, they were conducted before formal FE models. The first cross dependence was conducted to ensure that the cores sections in our data may highly influence the results of the regular FE or random effects (RE) results. Then, the authors applied the Hausman model specification test to specify the appropriate model for our dataset between the RE and FE. Consequently, the RE and the pooled OLS results are not reported with the aim of being precise and avoiding a lengthy redundant results part.

### 4.1 Descriptive statistics

**Fig. 1:**
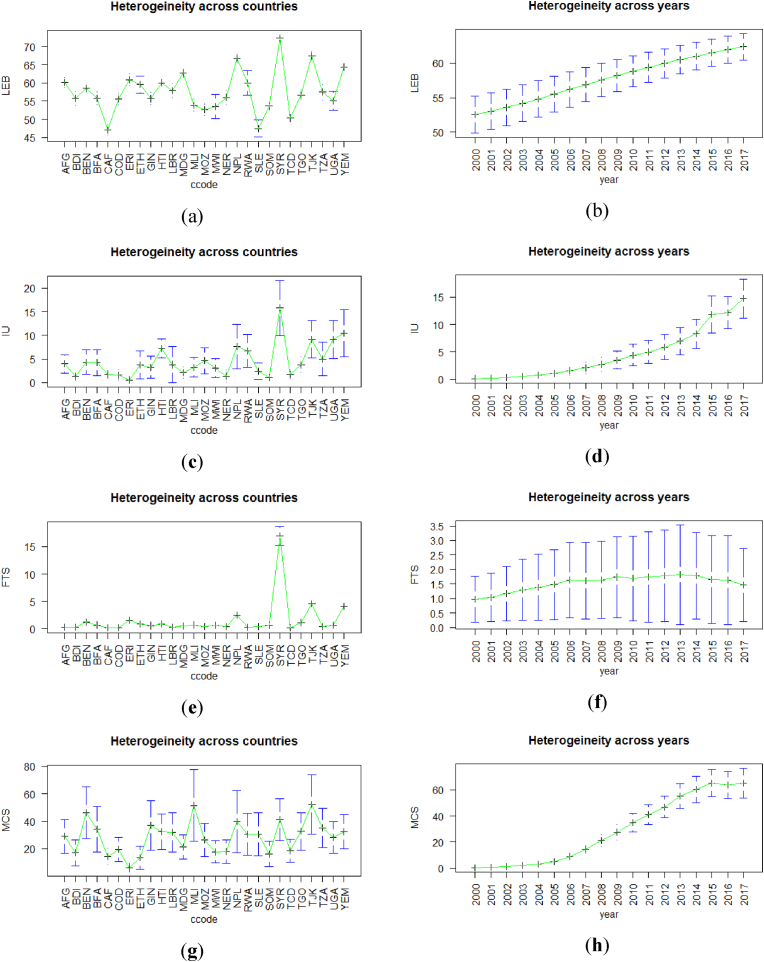
The figure of time and year heterogeneity for the dependent and the independent variables: LEB heterogeneity (a) across countries and (b) across years; IU heterogeneity (c) across countries and (d) across years; FTS heterogeneity (e) across countries and (f) across years; MCS heterogeneity (g) across countries and (h) across years.

Table 3 has the descriptive statistics of the variables. Looking at the mean, median, and the minimum of the dependent variables, the authors found that the numbers were low for low-income countries. On average, LICs' life expectancies were approximately 57 years of life. The minimum was 40 years of expected life. In Fig. 1, panels (a) and (b) show that the averages vastly differed for countries. In Fig. 1, it is noticeable that most countries’ average was between 50 and 65. Only three countries were below 50 throughout the whole-time span of our data from 2000 to 2017. Similarly, only three low-income countries were able to surmount the threshold of 65 years old. This indicates how low and severe LEB ratios are compared to higher-income countries which are around 80 years of expected life.

**Fig. 2:**
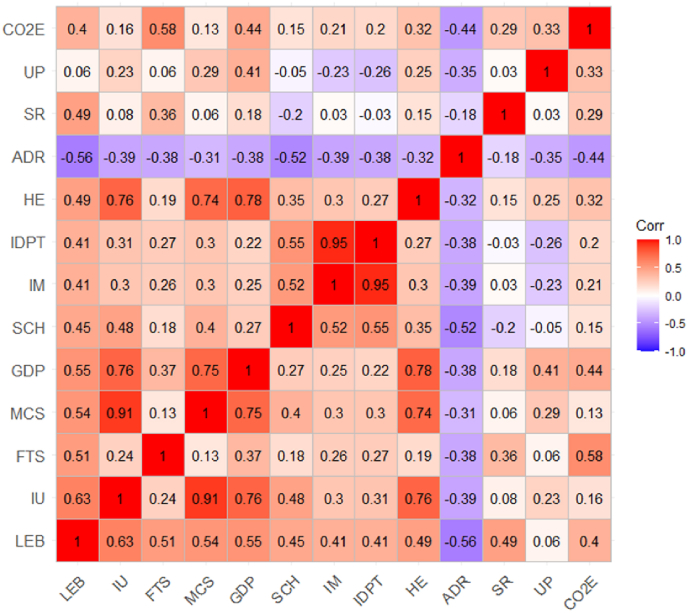
Spearman correlation matrix of the dependent health variables, independent ICT Variables, and socioeconomic and environmental control variables.

Similarly, Fig. 1 and Table 4 report the changes on average of our independent ICT variables. In this era of information technology, it is distressing that the average is 4% of the total population for low-income countries on internet use. It is noticeable that the 4.5% average indicates the across time and countries' average, and the internet trend started 20 years ago; therefore, to depict better the current country situation, Fig. 1 panel (b) shows the most recent average. Panel (b) in Fig. 1 shows that, in 2018, the average internet users was approximately 15% of the whole LICs’ population. As per the fixed-telephone subscriptions and mobile cellular subscriptions, the averages were 1.5% and 28.7%, respectively. Except for fixed-telephone subscriptions, an observer can see, in panels (b), (d), and (f) in Fig. 2, that internet use and mobile cellular subscriptions have amplified over the years. Of the ICT variables, mobile cellular subscriptions showed the most growth over time, and from 2016 to 2018, it averaged approximately 60% of the total population, see panel (f) in Fig. 1. Panels (a), (c), and (e) in Fig. 1 also show that the average user, for all ICT indicators, per year, differed from country to country, especially for internet users and mobile subscriptions.

Fig. 1 portrays cross-country heterogeneity and dependence for all variables dependent and independent. This type of heterogeneity may bias the results given by the estimation methods like the standard POLS. On the other hand, Fig. 2 presents the Spearman's correlation matrix for our set of variables. In this research, the advantage of this correlation matrix, needless to say: “not to ensure causality” is to indicate and describe a brief overview of the direction of the possible causality between our set of variables. Similar to the previous articles, the correlation matrix displayed that there may be a positive causal relationship. In other words, ICT scales and health outcomes presented through life expectancy correlated positively. Thus, health outcomes were possibly affected positively by ICT. The authors also observe in Fig. 2 that life expectancy correlated positively with other control variables, except for age dependency ratio (ADR). The previous literature and the findings in Fig. 2 display a rough idea of the causality direction between health variables and health inputs, and ICT.

Finally, in columns 1–4, Table 4, the first-generation tests results show the LLC and IPS panel unit root test results. Per the LLC method, the authors noticed that almost all variables were stationary at all levels, except for ADR and CO2E. As per the IPS method, not all variables were stationary-at-level. Even though some variables were not stationary-at-level, the authors took the sum of total stationarity cases as our role of thumb to decide to use static methods. Especially since the dependent variables are stationary at level, and only two of the three ICT variables are not stationary according to the IPS method. Still, it is a good suggestion that future estimation methods use dynamic models, incorporating the lag of the independent variables and maybe the lag of the non-stationary dependent variable. Under a stationarity assumption, the coming four sections will introduce the results of the cross-sectional estimation methods. After establishing the cross-sectional dependence, through the test illustrated in the next chapter and Fig. 2, the authors report the test results of second-generation tests (see Table 4: Second-generation panel unit root tests). The CIPS and CADF tests did not reject the assumption of cross-sections in the data. The second-generation test showed that almost all the results rejected the null hypotheses of the presence of panel unit roots. Therefore, the panel was stationary in both “drift” and “trend” settings.

### 4.2 Fixed-effects FE results

#### 4.2.1 One way (individual/country) fixed effects using Driscoll and Kraay robust errors (and with instrumental variables) estimation results

Table 5 reports the estimation results of fixed effects using Driscoll and Kraay robust errors (and with IV) estimation results, cross-dependence test results, and the Hausman model identification test results. Before using Driscoll and Kraay robust errors, cross dependence and Hausman model specification tests have been applied. All the tests’ results reject their null hypothesis and indicated that there was a cross-dependence effect in the data, and FE was superior to RE in estimating the model.

**Table 5:** Individual/Country FE using Driscoll and Kraay robust errors estimation results for Life Expectancy at Birth.

	Model			With IV		
	1	2	3	4	5	6
lnIU	0.017***			0.043***		
	(0.005)			(0.005)		
lnFTS		−0.006**			−0.080***	
		(0.003)			(0.016)	
lnMCS			0.014***			0.085***
			(0.004)			(0.023)
lnGDP	0.038***	0.074***	0.032***	−0.011	0.124***	−0.159**
	(0.008)	(0.011)	(0.005)	(0.012)	(0.027)	(0.062)
lnSCH	0.016**	0.029***	0.013***	−0.004	0.019*	−0.071***
	(0.007)	(0.008)	(0.004)	(0.011)	(0.010)	(0.021)
lnIM	−0.017*	−0.028**	−0.029*	0.004	0.001	−0.020
	(0.010)	(0.014)	(0.015)	(0.016)	(0.019)	(0.026)
lnIDPT	0.048***	0.065***	0.055***	0.022	0.063***	0.007
	(0.014)	(0.015)	(0.013)	(0.017)	(0.024)	(0.053)
lnHE	0.025***	0.038***	0.028***	0.0004	0.010	−0.037
	(0.008)	(0.006)	(0.007)	(0.010)	(0.014)	(0.026)
lnADR	0.219***	0.182***	0.192***	0.276***	0.180***	0.241***
	(0.048)	(0.054)	(0.049)	(0.037)	(0.068)	(0.039)
lnSR	6.887***	7.246***	7.606***	5.947***	4.383***	8.182***
	(1.144)	(1.308)	(1.056)	(1.061)	(1.156)	(1.515)
lnUP	0.070***	0.084***	0.075***	0.045**	0.045	0.017
	(0.017)	(0.016)	(0.014)	(0.023)	(0.028)	(0.032)
lnCO2E	−0.014**	−0.019***	−0.017***	−0.004	−0.009**	0.0004
	(0.005)	(0.004)	(0.004)	(0.008)	(0.003)	(0.005)
R2	0.820	0.785	0.813	0.750	0.517	0.491
Adj. R2	0.806	0.768	0.798	0.730	0.478	0.451
F-Stat	204.53***	164.16***	195.70***	1353.17***	538.08***	379.57***
	Breusch–Pagan LM test for cross-sectional dependence in panels					
	1601.3***	1561.2***	1496.3***			
	Pesaran CD test for cross-sectional dependence in panels					
	5.369***	7.088***	13.349***			
	Hausman Test					
	50.84 ***	50.80***	75.55***			

Significance level, *p < 0.1; **p < 0.05; ***p < 0.01. LEB is life expectancy at birth; IU is internet use; FTS is fixed telephones; MCS is mobile subscriptions; GDP is GDP per capita; SCH is school enrollment, secondary (% gross); IM is immunization against measles; IDPT is immunization against DPT; HE is Domestic general government health expenditure per capita, PPP (current inter ratio ln USD); ADR is age dependency ration; SR is sex ration; UP is urban population; CO2E is carbon oxide emissions; ICC is internet country code dummy; and CCSEB is communications, computer, etc. (% of service exports, BoP).

^1^Significance level, *p < 0.1; **p < 0.05; ***p < 0.01.

The fixed effects (FE) with Driscoll and Kraay robust errors results are in three columns (1–3). The IV settings results are in columns 3 to 6. In Table 5, the FE estimates show that two of the ICTs, IU, and MCS, were significant and had a positive effect on LEB. On the other hand, only one ICT, FTS, reduced LEB. In the FE, the IU estimate had the highest positive value of the other ICTs. The former passages imply that IU and MCS (FTS) estimators increase (decrease) LEB. Specifically, IU and MCS increased LEB by 1.7% and 1.4%, respectively; FTS reduced LEB by 0.4%. As per the results of FE with instrumental variables (IVs), the ICT variables changed their estimates. In Table 5, IU and MCS increased LEB by 4.3% and 8.5%, respectively; FTS reduces LEB by 8%. Now, the highest value through the IV set was the MCS estimate.

In both with and without IV settings, control variables’ estimates of the FE were also almost all significant. An observation in the control estimates reports that for all the FE (columns 1–3) models, GDP, SCH, IDPT, HE, ADR, SR, and UP increased LEB. In contrast, CO2E and IM decreased LEB. These results are consistent with previous research. In the IV settings (columns 4–6), the authors observed some changes in the significance, the degree, and the sign of our control estimates. In column 4, only SR, ADR, and UR remained statistically significant. In column 6, GDP, SCH, SR, ADR, and UR remained statistically significant; the statically significant estimates of GDP and SCH changed their signs. As per column 5, HE, IM, and UP became statistically insignificant.

#### 4.2.2 Two-way fixed effects using Driscoll and Kraay robust errors (and with instrumental variables) estimation results

Table 6 reports the results of the estimation of two-way fixed-effects estimators using robust Driscoll and Kraay. The two-way fixed-effects approach accounts for both country and time effects (it). Just as in FE Table 5, the two-way FE with Driscoll and Kraay robust errors (and with IV) results are reported in six columns, from column 1 to column 6. Table 6 also reports the cross-dependence and the Hausman model identification test results. Hence, the two tests’ results reject their null hypothesis. They indicate that (1) there was a strong cross-dependence effect on our data according to Breuch-Pagan cross-sectional dependence test; (2) the Pasaran CD test accepted the cross-sectional independence; (3) the two-way FE was superior to two-way RE in estimating our model.

**Table 6:** Two-way FE using Driscoll and Kraay robust errors estimation results for Life Expectancy at Birth.

	model			With IV		
	1	2	3	4	5	6
lnIU	0.003			−0.021***		
	(0.003)			(0.008)		
lnFTS		−0.004**			−0.018**	
		(0.002)			(0.009)	
lnMCS			0.002			−0.031
			(0.002)			(0.019)
lnGDP	0.021***	0.026***	0.021***	0.037***	0.037***	0.055**
	(0.003)	(0.005)	(0.003)	(0.012)	(0.008)	(0.023)
lnSCH	0.006	0.005	0.005	0.009	0.004	0.019
	(0.006)	(0.006)	(0.005)	(0.007)	(0.006)	(0.018)
lnIM	−0.035***	−0.037***	−0.037***	−0.063***	−0.033***	−0.067***
	(0.011)	(0.011)	(0.012)	(0.017)	(0.012)	(0.017)
lnIDPT	0.066***	0.068***	0.067***	0.087***	0.067***	0.092***
	(0.011)	(0.011)	(0.011)	(0.012)	(0.012)	(0.013)
lnHE	0.023***	0.023***	0.024***	0.032***	0.017**	0.030***
	(0.008)	(0.008)	(0.007)	(0.009)	(0.007)	(0.008)
lnADR	0.206***	0.202***	0.202***	0.169***	0.202***	0.198***
	(0.044)	(0.042)	(0.042)	(0.055)	(0.044)	(0.038)
lnSR	5.769***	5.635***	5.835***	5.690***	5.202***	4.451**
	(1.085)	(1.022)	(1.100)	(1.150)	(1.112)	(1.842)
lnUP	0.053***	0.051***	0.054***	0.054***	0.045***	0.041***
	(0.007)	(0.006)	(0.007)	(0.011)	(0.006)	(0.015)
lnCO2E	−0.006	−0.005	−0.006	−0.004*	−0.004	−0.006
	(0.004)	(0.003)	(0.003)	(0.002)	(0.003)	(0.004)
R-Squared:	0.365	0.369	0.364	0.228	0.315	0.189
Adj. R-Squared:	0.288	0.291	0.286	0.133	0.231	0.089
Chisq on 10 DF:	24.873***	25.252**	24.710***	190.588***	225.576***	167.710***
	Breusch–Pagan LM test for cross-sectional dependence in panels					
	2418.1***	2248.5***	2432.6***			
	Pesaran CD test for cross-sectional dependence in panels					
	−1.017	−1.568	−0.965			
	Hausman Test Results					
	60.37***	323.5***	58.24***			

Significance level, *p < 0.1; **p < 0.05; ***p < 0.01. LEB is life expectancy at birth; IU is internet use; FTS is fixed telephones; MCS is mobile subscriptions; GDP is GDP per capita; SCH is school enrollment, secondary (% gross); IM is immunization against measles; IDPT is immunization against DPT; HE is Domestic general government health expenditure per capita, PPP (current inter ratio ln USD); ADR is age dependency ration; SR is sex ration; UP is urban population; CO2E is carbon oxide emissions; ICC is internet country code dummy; and CCSEB is communications, computer, etc. (% of service exports, BoP).

Significance level, *p < 0.1; **p < 0.05; ***p < 0.01.

Correspondingly, the two-way fixed-effects (FE) with Driscoll and Kraay robust errors results are in three columns, from column 1 to column 3. The IV settings results are in three columns, from column 3 to column 6. In Table 6, the FE estimates showed that only FTS estimates, in both with and without IV setting, remain statistically significant. In addition, IU was only statistically significant in column 4 under the IV set. Hence, under both the settings, “FTS” reducedf LEB by 0.4% and 1.8%, respectively. Moereover, under the IV set, IU reduced LEB by 2.1%. The former passages imply that IU and FTS statistically significant estimates decrease LEB.

Control variables estimates of the two-way FE were also different to the individual FE part. For example, GPD, IM, IDPT, HE, ADR, SR, and UP were all statistically significant. On the other hand, neither SCH nor CO2E were statistically significant in both settings. Hence, CO2E was significant only in column 4.

### 4.3 Pooled and mean groups common correlated effects estimation results with TREND

In this part, Table 7 has the estimation results of both pooled and mean groups common correlated effects, “PCCE and MGCCE”, with “Trend”. In the presence of heteroskedasticity, serial and cross-sectional correlations, next to the Driscoll and Kraay robust errors, the authors of this paper also used the CCE. The proposed CCE estimator outperformed OLS in the presence of structural factors, and there was strong codependence in the data. Both CCE Table 7's results are in six columns, from column 1 to column 6. Correspondingly, The Pooled CCE results are in columns 1–3, and the mean groups CCE results are in columns 4 to 6.

**Table 7:** Pooled CCE and mean group CCE with robust errors estimation results for life expectancy at birth.

	Pooled			Mean Group		
	1	2	3	4	5	6
lnIU	0.001***			0.002***		
	(0)			(0)		
lnFTS		0**			0.004***	
		(0)			(0)	
lnMCS			0**			0.001***
			(0)			(0)
lnGDP	0	−0.002	0.004***	0.012***	0.002***	0.005***
	(0.002)	(0.002)	(0.001)	(0)	(0)	(0)
lnSCH	−0.002***	−0.002***	0	−0.011***	−0.015***	0***
	(0.001)	(0.001)	(0)	(0)	(0)	(0)
lnIM	0.006***	0.002	0.003***	−0.003***	0.002***	0.003***
	(0.002)	(0.001)	(0.001)	(0)	(0)	(0)
lnIDPT	0	0.004***	0	0.007***	0.002***	0.005***
	(0.003)	(0.001)	(0.001)	(0)	(0)	(0)
lnHE	0	0	−0.001***	0.006***	0.003***	−0.004***
	(0.001)	(0,.001)	(0)	(0)	(0)	(0)
lnADR	−0.019**	−0.045***	0.07	−0.007***	0***	0,001***
	(0.016)	(0.01)	(0.051)	(0)	(0)	(0)
lnSR	0.019	−0.001	0.297***	0***	0***	0***
	(0.299)	(0.622)	(0.027)	(0)	(0)	(0)
lnUP	0.053***	0.051***	0.001	0.004***	0.001***	0.001***
	(0.02)	(0.013)	(0.012)	(0)	(0)	(0)
lnCO2E	0.001***	0.001***	0.001***	−0.006***	0.002***	0.002***
	(0)	(0)	(0)	(0)	(0)	(0)
Total Sum of Squares:	6.591	6.591	6.591	6.591	6.591	6.591
Residual Sum of Squares:	0.001	0.001	0	0	0	0
Multiple R-squared:	0.999	0.999	1	1	1	1

Significance level, *p < 0.1; **p < 0.05; ***p < 0.01. LEB is life expectancy at birth; IU is internet use; FTS is fixed telephones; MCS is mobile subscriptions; GDP is GDP per capita; SCH is school enrollment, secondary (% gross); IM is immunization against measles; IDPT is immunization against DPT; HE is Domestic general government health expenditure per capita, PPP (current inter ratio ln USD); ADR is age dependency ration; SR is sex ration; UP is urban population; CO2E is carbon oxide emissions; ICC is internet country code dummy; and CCSEB is communications, computer, etc. (% of service exports, BoP).

The results of this section are different from the results in Table 5, Table 6. Table 7, in both the PCCE and MGCCE settings, were positive and statistically significant. The first remark is that although all the independent ICT variables are statistically significant, yet, they all are less than 0.5% or close to zero. According to the PCCE and MGCCE, IU, MCS, and FTS estimators had a small to almost negligible effect on increasing LEB. More specifically, in the PCCE setting, IU, MCS, and FTS increase LEB by 0.1%, 0%, and 0%, respectively. While in the MGCCE model, the results of IU, MCS, and FTS estimators increased LEB by 0.4%, 0.2%, and 0.1%, respectively.

In Table 7, the control variables’ estimates of the PCC models are also different than the FE models. All the control variables were statistically significant in the MGCCE in columns 4–6. As per the PCCE model, some controls were statistically significant in some columns. For example, in column 1, the statistically significant ones were SCH, IM, ADR, UP, and CO2E. Hence, in column 2, the statistically significant ones were SCH, IDPT, ADR, UP, and CO2E. Finally, in column 3, the statistically significant ones were GDP, IM, HE, SR, and CO2E. The signs and degree of significance of the PCCE and MGCCE differ from the previous ones found in both Table 5, Table 6 of the FE estimates.

### 4.4 Feasible generalized least squares estimation results

In this part, Table 8 has the estimation results of feasible generalized least squares. The FGLS Table 8 results are reported in three columns, from column 1 to column 3. In the presence of heteroskedasticity, serial, and cross-sectional correlations, next to the Driscoll and Kraay robust errors, the FGLS. the proposed FGLS estimator outperformed OLS by consistently estimating the large error covariance matrix. In Table 8, even though the IU estimate was not statistically significant, but the other two estimates of ICT indicators FTS and MCS were. While FTS was negative, MCS was positive. The former passage implies that FTS (MCS) estimates decreased (increase) LEB, respectively. More specifically, FTS (MCS) reduced (increased) LEB by 0.2% (1%), respectively.

**Table 8:** Feasible generalized least squares estimation results for LEB.

	lnLEB		
	1	2	3
lnIU	0.002		
	(0.002)		
lnFTS		−0.002*	
		(0.001)	
lnMCS			0.01***
			(0)
lnGDP	0.021***	0.045	0.018***
	(0.003)	(0.003)	(0.003)
lnSCH	0.005	0.017***	0.008***
	(0.005)	(0.002)	(0.001)
lnIM	−0.037***	−0.013***	−0.011**
	(0.012)	(0.005)	(0.005)
lnIDPT	0.067***	0.037***	0.026***
	(0.011)	(0.005)	(0.004)
lnHE	0.024***	0.021***	0.014***
	(0.007)	(0.002)	(0.002)
lnADR	0.202***	0.02	0.058**
	(0.042)	(0.033)	(0.029)
lnSR	5.835***	4.829***	5.209***
	(1.1)	(0.826)	(0.65)
lnUP	0.054***	0.072***	0.052***
	(0.007)	(0.012)	(0.012)
lnCO2E	−0.006	−0.007***	−0.007***
	(0.003)	(0.001)	(0.001)
Total Sum of Squares:	6.590	6.590	6.590
Residual Sum of Squares:	0.721	0.611	0.525
Multiple R-squared:	0.890	0.907	0.920

Significance level, *p < 0.1; **p < 0.05; ***p < 0.01. LEB is life expectancy at birth; IU is internet use; FTS is fixed telephones; MCS is mobile subscriptions; GDP is GDP per capita; SCH is school enrollment, secondary (% gross); IM is immunization against measles; IDPT is immunization against DPT; HE is Domestic general government health expenditure per capita, PPP (current inter ratio ln USD); ADR is age dependency ration; SR is sex ration; UP is urban population; CO2E is carbon oxide emissions; ICC is internet country code dummy; and CCSEB is communications, computer, etc. (% of service exports, BoP).

As per the other control variables, in Table 8, almost all of these variables remain statistically significant. Only the SCH and CO2E (GDP and ADR) estimates were not statistically significant for column 1 (column 2).

## 5 Discussions

In this study and from the prior and posterior tests (panel unit root tests, cross-sectional dependence tests, Hausman test, etc), the observer may first note that the setting of this work's data differed from previous similar pieces of research. The dataset presented in this research was stationary and cross-sectionally dependent. To remedy this difference, the authors sought to use different estimation methods. Precisely, this difference had the consequence of using results adequate for the context of our data (i.e., Driscoll and Kraay robust estimates, common correlated effects estimates, and feasible generalized least squares.). Table 5, Table 6, Table 7, Table 8 show the results of the estimation methods (individual fixed-effects FE (and with IV), two-way fixed-effects FE (and with IV), and pooled and mean group (CCE and FGLS).

The first observation from this research report is the apparent difference in the theoretical approach to estimating the causal effect. This paper is the only one to address the cross-sectional dependence in the estimation both in the presence of common factors and correlation in the error terms. Hence, in the following parts, the authors give a detailed discussion of the differences and consistencies in the group of countries level.

### 5.1 Fixed effects with/without instrumental variables

The first model discussed is the Individual FE presented in Table 5. Individual FE estimation results show that two of the ICT variables “IU and MCS” had a significant and positive effect on raising LEB. On the other hand, the FTS of our ICT variable had a significant negative effect on LEB. To discuss the findings appropriately, they were compared and assessed with similar pieces of research. It was found that the individual FE results were close to some results of the previous articles and different from others. Almost all of our IU, MCS individual FE (with IV) results were close to the estimation results of Majeed and Khan, Alhassan and Adam, Văidean and Achim, and Karaman Aksentijević et al. on more than a hundred different income countries. This model's findings also resemble Adeola and Evans, Mimbi and Bankole, and Bhusal and Ghimire African countries, Jing et al. 18 Asian countries (Adeola & Evans, 2018; Alhassan & Adam, 2021; Bankole & Mimbi, 2016; Bhusal, 2022; Jing et al., 2022; Karaman Aksentijević et al., 2021; Lee et al., 2016; Majeed & Khan, 2019; Văidean & Achim, 2022). In contrast, all our FTS Individual FE (and with IV) results are different from the estimation results of the previously mentioned authors.

### 5.2 Two-way fixed effects with/without instrumental variables

As per both two-way FE with/without IVs in Table 6, the estimation results were different from the previous ones. The two-way FE results show that two of our three ICT variables (IU and MCS) have a positive but not significant effect on LEB. On the other hand, it is shown that first, all ICT variables are negative in two-way FE with IV, and second only FTS and MCS are significant. Hence, FTS was significant and negative in both settings, which resulted in a negative effect on LEB. To debate the findings appropriately, they were compared with similar papers. The comparisons showed that the two-way FE with/without IVs' results are different from almost all the results of Majeed and Khan, Alhassan and Adam, Văidean and Achim, and Karaman Aksentijević et al. on more than a hundred different-income countries. This model's findings also differ from the results found in African and Asian countries, presented by Adeola and Evans, Mimbi and Bankole, and Jing et al. (Adeola & Evans, 2018; Alhassan & Adam, 2021; Bankole & Mimbi, 2016; Bhusal, 2022; Jing et al., 2022; Karaman Aksentijević et al., 2021; Lee et al., 2016; Majeed & Khan, 2019; Văidean & Achim, 2022). In contrast, our two-way FE with/without IV results only resembled the estimation results of Bhusal and Ghimire in African countries (Bhusal, 2022).

### 5.3 Pooled and mean group common correlated effects

On the other hand, in Table 7, the PCCE and MGCCE, the estimation results showed that all our three ICT variables “IU, FTS, and MCS” had a significant and positive effect on raising LEB. The same as the previous two parts, to assess these findings appropriately, they are compared with similar pieces of research. Hence, the PCCE and MGCCE results were close to almost all of the models presented in the previous articles on different-income, African, and Asian countries. Previous articles' results, which are not significant or positive, have the apparent difference. Some differences were the results presented in Majeed and Khan on different income countries (Majeed & Khan, 2019) and Bhusal and Ghimire on African countries (Bhusal, 2022).

### 5.4 Feasible generalized least squares

Finally, the FGLS estimation results were also different than the previous parts. The estimation outcomes showed that IU, FTS, and MCS had only positive, negative and significant, and a positive and significant effects on LEB, respectively. It is found that the FGLS results are only similar to some models' results from the previous articles. On the other hand, they are different from the previous models’ results on Asian, African, and different-income countries (Adeola & Evans, 2018; Alhassan & Adam, 2021; Bhusal, 2022; Jing et al., 2022; Karaman Aksentijević et al., 2021; Lee et al., 2016). The only similarities are found in the estimation results of mobile subscriptions of the following: Majeed and Khan; Văidean and Achim; Mimbi and Bankole; Alhassan and Adam (Alhassan & Adam, 2021; Bankole & Mimbi, 2016; Majeed & Khan, 2019; Văidean & Achim, 2022).

In this paper, the authors aimed to discuss and empirically address the dilemma that low-income countries face concerning ICT and health. Almost all our fixed-effects (both individual/country effects with/without IVs), pooled, and mean group CCE and FGLS results suggest that mobiles and internet use play an important role in increasing life expectancies, thus enhancing low-income countries’ health outcomes. Meanwhile, telephone subscriptions (FTS) are shown in the fixed-effects (both individual/country effects and two-way effect with/without IVs) and FGLS results to reduce the life expectancy of our 27 low-income countries. The previous statement means that the usage of fixed phones is becoming obsolete and antiquated. More precisely, our results show strong evidence of the role of the internet era through utilizing mobiles to better the health systems of low-income countries. Yet, all our significant results still were significantly small, especially in the case of the common correlated effects. This indicates the purpose of the study that “more ICT may not result in significantly more life expectancies” for low-income countries.

While our research aimed to study the causality of ICT on health, it encountered some limitations in the form of future research directions. For example, formulating new policies and strategies was not sufficient due to the quantitative nature of this study. Researchers should consider a qualitative study sort to deduce more findings. Qualitative research will further inspect ICT's impact on bridging the health iniquities gap and general healthy living standards. Life expectancy at birth only considers the number of expected years but measures neither the future quality of life nor chronic diseases. In addition, the data were limited to the healthcare provided by the government sector. Hence, future studies should consider a more comprehensive panel data that measures expected life quality and a measurement variable that includes the private sector, and addresses the issue of mental health (e.g., stress, depression, and anxiety). Generally, broader health variables that consider maternal health, chronic and different diseases, mental health, and the quality of life should be viewed by future studies.

The use of principal component analysis (PCA) to reduce possible collinearity between our ICT variables for these LICs, and study this impact on other health outcome variables. This paper's research analysis was not a dynamic design, and the results were static and interpreted short-term dependency. Future research could use spatial dynamic panel models. Once programmed, Shi's (Shi & Lee, 2017) model may serve as an example. And it is suggested, in the least, the use of Dynamic System and Difference Generalized Method of Moments to deal with the afore-mentioned design issues.

Finally, research on LICs is scarce in the direction of current innovation-based deployment of digitalization, artificial intelligence, and big data in sustainable development (Di Vaio, Hassan, & Alavoine, 2022; Leal Filho et al., 2022), which could be the aim of future works.

## 6 Conclusions

In conclusion, the authors studied nexus between ICT and LICs' health outcomes. While the literature has suggested a positive relationship between ICT and health outcomes (Adeola & Evans, 2018; Alhassan & Adam, 2021; Bankole & Mimbi, 2016; Bhusal, 2022; Jing et al., 2022; Karaman Aksentijević et al., 2021; Khelfaoui et al., 2022; Lee et al., 2016; Majeed & Khan, 2019; Văidean & Achim, 2022), the authors in this study expanded the literature by empirically examining the causality between ICT and the LICs' health. Methodologically, the study provided new insights into the cross-sectional dependence of ICT indicators and health. For this endeavor, the authors utilized novel cross-sectional models and endogeneity treatments. The main findings claim that the internet and mobiles enhanced the health response of developing countries. On the contrary, telephone-based ICT decreases their health. For practical implications, this study's findings have many relevant insinuations for policymakers. In this internet era, many LICs have begun to work on sustainable healthcare delivery. LICs' health sectors should be founded on digital health. The combined use of the internet and mobiles for research, data acquisition, surveillance, access to patient data, and clinical care could be used to improve the capability of health workers, particularly in resource-limited settings in LICs. Given this, policymakers and private actors in the health industry must work together to build regulations that boost the performance of ICT at all levels of healthcare in LICs.

## Ethical approval and consent to participate

Not applicable.

## Funding

This work was supported by the 10.13039/501100001809National Natural Science Foundation of China (Grant No. 72274116), Guangdong Basic and Applied Basic Research Foundation (Grant No. 2021A1515011599), STU Scientific Research Initiation Grant (Grant No. STF20012) and Open Fund of Key Research Base of Philosophy and Social Science of Higher Education in Guangdong Province-Local Government Development Research Institute of 10.13039/100009047Shantou University (Grant No. 07419005 and 07421005).

## Consent for publication

Not applicable.

## Availability of supporting data

The datasets used and/or analyzed during the current study are available from the corresponding author upon reasonable request.

## CRediT authorship contribution statement

**Wenxin Wang:** Supervision, Project administration, Funding acquisition. **Issam Khelfaoui:** Writing – original draft, Visualization, Software, Resources, Methodology, Investigation, Formal analysis, Data curation, Conceptualization. **Danish Ahmed:** Writing – review & editing, Validation, Methodology, Investigation, Formal analysis. **Yuantao Xie:** Writing – review & editing, Validation, Supervision, Methodology, Conceptualization. **Muhammad Hafeez:** Writing – review & editing, Methodology, Formal analysis, Conceptualization. **Hicham Meskher:** Writing – review & editing, Visualization.

## Declaration of competing interest

The authors declare that they have no known competing financial interests or personal relationships that could have appeared to influence the work reported in this paper.

## Data Availability

Data will be made available on request.
